# Microbial Biogeography of Arctic Streams: Exploring Influences of Lithology and Habitat

**DOI:** 10.3389/fmicb.2012.00309

**Published:** 2012-08-24

**Authors:** Julia R. Larouche, William B. Bowden, Rosanna Giordano, Michael B. Flinn, Byron C. Crump

**Affiliations:** ^1^Rubenstein School of Environment and Natural Resources, University of VermontBurlington, VT, USA; ^2^Institute of Natural Resource Sustainability, Illinois Natural History Survey, University of Illinois at Urbana–ChampaignIL, USA; ^3^Biological Sciences, Murray State UniversityMurray, KY, USA; ^4^Horn Point Laboratory, Center for Environmental Science, University of MarylandCambridge, MD, USA

**Keywords:** arctic streams, bacterial community composition, microbial community composition, lithology, T-RFLP, 16S rRNA gene sequencing

## Abstract

Terminal restriction fragment length polymorphism and 16S rRNA gene sequencing were used to explore the community composition of bacterial communities in biofilms on sediments (epipssamon) and rocks (epilithon) in stream reaches that drain watersheds with contrasting lithologies in the Noatak National Preserve, Alaska. Bacterial community composition varied primarily by stream habitat and secondarily by lithology. Positive correlations were detected between bacterial community structure and nutrients, base cations, and dissolved organic carbon. Our results showed significant differences at the stream habitat, between epipssamon and epilithon bacterial communities, which we expected. Our results also showed significant differences at the landscape scale that could be related to different lithologies and associated stream biogeochemistry. These results provide insight into the bacterial community composition of little known and pristine arctic stream ecosystems and illustrate how differences in the lithology, soils, and vegetation community of the terrestrial environment interact to influence stream bacterial taxonomic richness and composition.

## Introduction

The underlying lithology of watersheds controls the physical structure of landscapes, but also influences their biology by controlling the chemistry of soils (Jenny, 1980), plants (Whittaker, 1960), and water (Hynes, 1975). The composition of bacterial communities may also be sensitive to factors controlled by underlying lithology, particularly in arctic stream ecosystems where water chemistry has been shown to influence the composition of epiphyte and macroinvertebrate communities (Slavik et al., 2004). The few studies that are available demonstrate environmental controls on the variability in stream bacterial communities related to stream water pH (Fierer et al., 2007), organic matter sources (Van Hannen et al., 1999), and available nutrients such as the dissolved forms of organic carbon and inorganic nitrogen and phosphorus (Findlay and Sinsabaugh, 2006). These types of studies guide our understanding of first order controls on community composition, but we have yet to identify whether microbial composition in streams is linked to overarching, watershed controls such as lithology.

Sessile bacteria are ecologically important members of the biota in streams and other aquatic environments. Microbial community composition in aquatic ecosystems are responsive to many different chemical and biological factors including physical variables (i.e., temperature variations, climate, topography, and light availability; Kaplan and Bott, 1989; Autio, 1998) and biogeochemical variables (i.e., the quality and quantity of carbon sources, inorganic nutrients, and electron acceptors; Drever, 2002; Crump et al., 2003; Eiler et al., 2003). Recent studies have also shown that bacterial communities within biofilms are important regulators of stream biogeochemical functions (Sobczak and Findlay, 2002; Hall et al., 2009) and have the potential to generate unique biogeochemical signatures across stream types.

These previous studies suggest, therefore, that the microbial community composition in streams could be influenced by biogeochemical cues from the local landscape but could in turn strongly influence the biogeochemical characteristics observed in streams. It is likely that both of these alternatives interact to varying degrees in different ecosystems, but it would be difficult to test these alternatives in a field study. Nevertheless, few studies have investigated the effect of combined lithological and biogeochemical differences on attached microbial community structure between streams (Takai et al., 2003; Skidmore et al., 2005; Oline, 2006) or the effect of habitat differences within streams (Hullar et al., 2006). This study is targeted to a unique environment where lithology differs over a small-scale and is likely to have a strong influence on the community structure of sessile bacteria, providing the opportunity to detect biogeographical patterns.

We hypothesized that fundamentally different lithologies that support significantly different vegetation communities on land also support significantly different bacterial communities in streams by imparting different biogeochemical characteristics to water, which could be further modified by the lithology-specific bacterial communities. Our objectives were: (1) compare stream bacterial community composition among streams emerging from the three distinctly different lithologies (non-carbonate, NC; complex sedimentary, CS; and ultramafic, UM) that dominate the Noatak National Preserve region of arctic Alaska; (2) compare stream bacterial community composition among two different stream habitat types (sediment vs. epilithon); and (3) determine if community composition patterns correlate with the biogeochemical characteristics of streams for the three different lithologies. While we expected to find differences at the microhabitat scale due to variations in resources (e.g., light availability and organic matter sources) and hydrologic stressors, we were most interested in detecting differences across lithologies, which may suggest a sensitivity of microbial community composition at the broader, landscape scale. This work adds to a small but growing base of knowledge about microbial biogeography in arctic ecosystems that are currently responding to a rapidly changing climate (Crump et al., 2003, 2007; Skidmore et al., 2005; Galand et al., 2006; Garneau et al., 2006).

## Materials and Methods

The Noatak River is in the Noatak National Preserve in Alaska (USA). It is the longest continuous river in the USA National Wild and Scenic Rivers system and the largest mountain-ringed river basin in North America, virtually unaltered by direct and indirect (e.g., nitrogen deposition) human activity (Milner et al., 2005). The lithology of this area is complex (Jorgenson et al., 2002) but includes three important and distinctly different types that were the focus of this study. Ultramafic rocks (basalt, gabbro, peridotite, pyroxenite, dunite) of the Siniktanneyak mountains tend to be high in iron and magnesium with sparse vegetation. Non-carbonate rocks (glaciolacustrine deposits, conglomerate, sandstone, shales) of the Avingyak Hills support acidic, organic-rich soils, and host shrub birch, willow and ericaceous plants. Complex sedimentary rocks (shale, basalt, limestone, and mafic rocks) of the Aniuk mountains support vegetation similar to the non-carbonate lithology. Jorgenson et al. (2002) found that the composition of plant communities differed by lithology, possibly due to variations in soil pH and phytotoxic effects of soluble minerals. Jorgenson et al. (2009) showed NC soils contain higher available soil phosphorus compared to UM soils, suggesting an interaction between vegetation and soils that may influence the nutrient content of soil water that subsequently enters streams.

**Figure 1:**
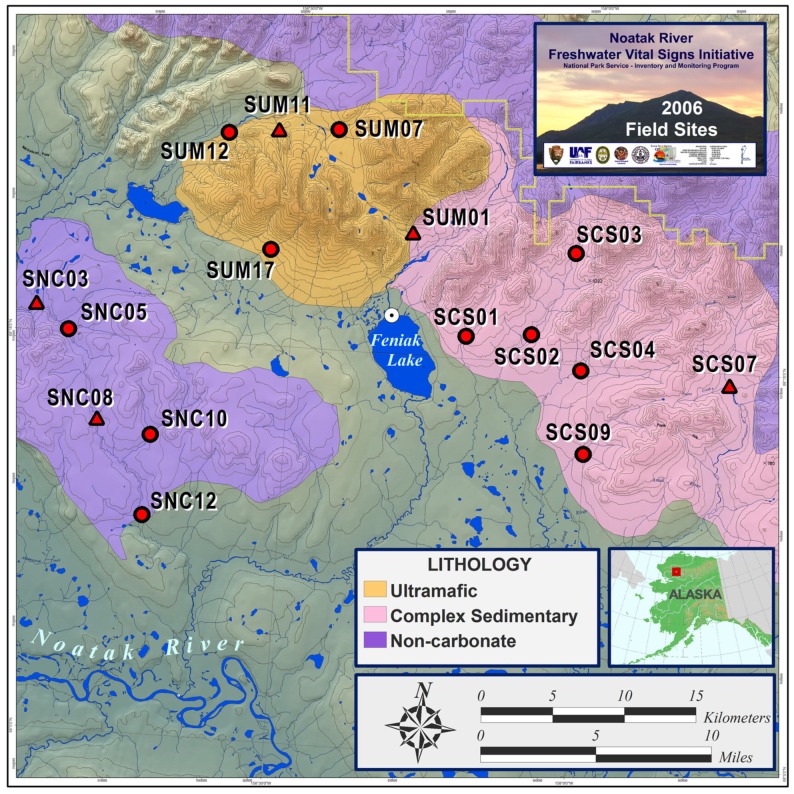
**Study area of Feniak Lake region with stream site locations across contrasting lithologies in the Noatak National Preserve, Alaska (Map credit: Andrew Balser)**. T-RFLP analyses were performed on sediment samples at all sites and epilithon samples at a subset of sites. 16S clone libraries were built from sediment and epilithon samples from sites indicated with triangles.

Samples were collected between July 8 and July 13, 2006 from headwater stream tributaries arising from catchments with uniform and contrasting lithologies. We sampled 15 streams located in the foothills of the Delong Mountains on the northern edge of the Noatak River basin in the vicinity of Feniak Lake (68°14′56.55″N and 158°19′19.90″W, elevation 1,411 feet). Terminal Restriction Fragment Length Polymorphism (T-RFLP) analyses were performed on all streams and 16S clone libraries were constructed from both sediment and epilithon samples from five sites (Figure 1).

Replicate first or second-order stream reaches were sampled within each separate lithology: four non-carbonate, five ultramafic, and six complex sedimentary (Figure 1). Sediment samples were collected in triplicate along a 25-m reach of each stream. Single epilithon (rock scrub) samples were also collected from cobble-bottom streams, but only from ultramafic and non-carbonate lithologies. Stream water chemistry sampling of the water column took place at the same time as microbial sampling and analysis details are provided in Flinn et al. (2009). Stream biogeochemical variables mentioned in this paper are defined as ${\text{NO}}_{\text{3}}^{-}$ (nitrate); TDN (total dissolved nitrogen); TDP (total dissolved phosphorus); TDN-${\text{NO}}_{\text{3}}^{-}$ (total dissolved nitrogen minus nitrate = indication of dissolved organic nitrogen, DON); ${\text{NO}}_{\text{3}}^{-}$/TDN (nitrate as a proportion of total dissolved nitrogen); TDN/TDP (ratio of total dissolved nitrogen to total dissolved phosphorus); DOC (dissolved organic carbon); and cations (calcium, Ca^2+^; magnesium, Mg^2+^; potassium, K^+^; and sodium, Na^+^).

Surface (∼3 cm deep) sediment samples for microbial analysis were collected in sterile 15-ml plastic tubes, preserved immediately with sucrose lysis buffer [SLB; 20 mM EDTA, 400 mM NaCl, 0.7 M sucrose, 50 mM Tris (pH 9.0)] in a 1:1 ratio, frozen on dry ice in the field, and stored at −80°C in the laboratory until analysis. Epilithic material from the tops of six submerged rocks in riffle sections of each stream reach was scrubbed with a nylon brush, rinsed into a sterile plastic container with filtered (0.22 μm) stream water, combined, and collected in a 0.22-μm Sterivex filter capsule (Millipore). Filter capsules were removed from syringes, flooded with 1 ml DNA extraction buffer [100 mM Tris (pH 8), 100 mM NaEDTA (pH 8), 100 mM phosphate buffer (pH 8), 1.5 M NaCl, 1% CTAB], frozen immediately on dry ice in the field, and stored at −80°C in the laboratory until analysis.

DNA extractions used the MoBio Power Soil DNA extraction kit (MoBio Laboratories, Inc., Carlsbad, CA, USA) following the manufacturer’s protocol with the following modification: a FastPrep Homogenizer and Isolation System (Thermo Fisher Scientific Waltham, MA, USA) was used to shake the MoBio extraction tubes at 4.5 m/s for 30 s to ensure complete cell lysis of bacteria. Extractions were conducted on 500-μl subsamples of streambed sediment (1:1 sediment:SLB slurry), and on filters of the rock biofilm samples that were removed from the Sterivex capsules.

Terminal restriction fragment length polymorphism analysis was conducted on all sediment (42 total) and epilithon samples (eight total). The 16S rRNA gene was amplified using Illustra PuReTaq Ready-To-Go PCR Beads (GE Healthcare Life Sciences, Piscataway, NJ, USA), 2 μl of DNA, and the following primers: HEX-labeled primer Bac8f and unlabeled primer Univ1492r (Reysenbach and Pace, 1995) obtained from Sigma-Genosys (St. Louis, MO, USA) and Invitrogen (Carlsbad, CA, USA), respectively. The PCR protocol consisted of an initial denaturation at 94°C for 4 min, followed by 94°C for 45 s, 54°C for 20 s, and 72°C for 2.5 min for 40 cycles, and a final 4 min at 72°C. Two separate PCR reactions for each DNA sample were pooled and digested separately in triplicate with three restriction enzymes: *Msp*I, *Alu*I, and *Hin*P1I (New England BioLabs, Beverly, MA, USA). Restriction digests (25 μl) consisted of 10 μl PCR product, 1 unit enzyme, 2 μl of 10× reaction buffer 2 (New England BioLabs), and sterile Sigma water (Sigma-Aldrich). PCR products were digested overnight at 37°C. Fluorescently labeled terminal restriction fragments (T-RFs) were size separated on an ABI Avant Genetic Analyzer 3100 (Applied Biosystems, Foster City, CA, USA) using an internal size standard (BioVentures MapMarker 1000, BioVentures, Inc., Murfreesboro, TN, USA).

Terminal restriction fragment length polymorphism electropherograms were analyzed using GeneMapper software version 3.7 (Applied Biosystems, Foster City, CA, USA). T-RF peaks that differed by less than 0.5 bp were grouped (Dunbar et al., 2001), and peaks > 80 bp with >50 relative fluorescent units were included in the analysis. Triplicate profiles were collapsed into one average profile by including peaks that occurred in two of the three replicate profiles in order to eliminate false peaks arising from dust or bubbles present in the capillaries of the detector. T-RFs of different lengths represent distinct operational taxonomic units (OTUs) but should not be interpreted as specific bacterial species because similar restriction fragment sizes can be produced from different organisms (Liu et al., 1997).

Terminal restriction fragment length polymorphism statistical analyses were performed in DECODA (Database for Ecological Community Data) version 3 (Minchin, 1990). Non-metric multidimensional scaling (NMS; Clarke, 1993) was used for ordination of the T-RFLP data using T-RF length and normalized peak height from all three restriction enzymes as input data. Similarities between samples were based on the Bray–Curtis dissimilarity matrix (Bray and Curtis, 1957), which has been recommended (Rees et al., 2004) and commonly used for T-RFLP data (Denaro et al., 2005; Deslippe et al., 2005; Fierer et al., 2007). Analysis of Similarity (ANOSIM; Clarke and Green, 1988; Clarke, 1993) was used to determine which samples were most closely related with patterns of similarity between bacterial communities using the Gower metric (Gower, 1971). Vector analysis was used to examine correlations between microbial community patterns and corresponding environmental data.

Representative samples with the highest degree of variation using T-RFLP were chosen for more detailed phylogenetic analyses. Clone libraries were prepared with the PCR-amplified 16S rRNA gene from sediment (*n* = 5) and epilithon (*n* = 4) from representative streams within the non-carbonate (two streams), ultramafic (two streams), and complex sedimentary lithologies (one stream, sediment only). The 16S rRNA gene was amplified using primers Bac8f (unlabeled) and Univ1492r (Invitrogen) with the protocol: 94°C for 2 min, followed by 40 cycles of 94°C for 30 s, 54°C for 20 s, and 72°C for 1.5 min with a final extension of 15 min at 72°C. To minimize the effects of PCR drift, PCRs were run in triplicate and pooled for each DNA extract. PCR products were run on 0.75% agarose gels, visualized with ethidium bromide, excised with a sterile razor blade, purified with Zymoclean Gel DNA Recovery kit (Zymo Research, Orange, CA, USA), cloned into pCR^®^2.1 vector using the TA cloning kit (Invitrogen), and transformed into OneShot^®^ Competent Cells (Invitrogen). Transformants were plated on Luria broth (LB) agar medium containing ampicillin, X-gal, and isopropyl-β-d-thiogalactopyranoside (IPTG). Ampicillin-resistant and β-galactosidase-negative clones were randomly selected and grown overnight at 37°C in LB with ampicillin. Clones were tested for the presence of the 16S rRNA gene inserts by PCR amplification using modified M13 primers: M13Long Forward (5′-CAGGAAACAGCTATGACCATGATTAC-3′) and M13Long Reverse (5′-GTAAAACGACGGCCAGTGAATTGT-3′) designed to the pCR^®^2.1 vector. An excess of 100 clones for each sample were sequenced using the M13 primer listed above as well as internal 16S primers custom designed for specific clone groups in this study to ensure complete overlap of sequence reads in both directions: 16S-A1F (5′-GTGCCAGCAGCCGCGGTAATAC-3′); 16S-A1R (5′-GTATTACCGCGGCTGCTGGCAC-3′); 16S-B1F (5′-GGTGCTGCATGGCTGTCGTCAGC-3′); 16S-B1R (5′-GCTGACGACAGCCATGCAGCACC-3′); 16S-B2F (5′-GGTGGTGCATGGTTGTCGTCAGC-3′); and16S-B2R (5′-GCTGACGACAACCATGCACCACC-3′). Clones from SedSNC03 were sequenced using the following protocol: 96°C for 1 min, followed by 25 cycles of 96°C for 10 s, 50°C for 5 s, and 60°C for 4 min. Ready to load sequence reactions were run at Vermont Cancer Center, University of Vermont on an ABI Avant Genetic Analyzer 3100 (Applied Biosystems). PCR products of the other eight clone libraries were cleaned and sent to Agencourt Bioscience Corporation (Beverly, MA, USA) for sequencing.

Clone sequences were assembled and edited using Sequencher version 4.6 (Gene Codes, Ann Arbor, MI, USA) aligned using the Ribosomal Database Project II (RDP) release 9.58 web resource^1^ (Brown, 2000; Gutell et al., 2002; Cole et al., 2007), and checked for chimeric sequences using the RDP’s CHIMERA_CHECK program based on the Pintail algorithm (Ashelford et al., 2005), Chimera Slayer in the Mothur software package (Schloss et al., 2009), and through manual inspection of aligned sequences in the software package ARB (Ludwig et al., 2004). Twenty-seven chimeric sequences were removed. Sequences were then classified with the RDP Classifier (Wang et al., 2007) and 123 chloroplast sequences (mainly from diatoms) and 11 mitochondria sequences were removed. The remaining 625 sequences were clustered into OTUs (97% sequence similarity) using uclust in the QIIME software package (Caporaso et al., 2010). Sequences have been submitted to GenBank under accession numbers FJ849067–FJ849648.

## Results

In general, study streams were typical ultraoligotrophic foothill tundra and mountain streams. Mountain streams were dominant in the UM lithology, characterized by mountain runoff, unstable substrate, scoured channels, and sparse biota. Tundra streams were dominant in the NC lithology, characterized by tundra runoff, organic peat-lined banks, and moderate biota. All streams were similar in physical structure and several basic water quality parameters including pH (7.3–7.8) and water temperature ranges (5.5–12°C; Table 1). Complex sedimentary streams had high electrical conductivity (502 ± 140 μS/cm^2^) compared to UM and NC streams (40 ± 5 and 168 ± 26 μS/cm^2^, respectively). Non-carbonate streams had higher concentrations of some metals (e.g., Al, Fe, and Si), DOC, and TDN. Ultramafic streams had higher concentrations of ${\text{NO}}_{\text{3}}^{-}$ compared to NC streams resulting in nearly a 10-fold higher proportion of ${\text{NO}}_{\text{3}}^{-}$ to TDN (${\text{NO}}_{\text{3}}^{-}$/TDN).

**Table 1:** **Values of biological, chemical, and physical parameters for study streams by lithology (CS, Complex Sedimentary; NC, Non-carbonate; UM, Ultramafic)**.

Parameters	**Lithology**		
	CS ± 1 SE	NC ± 1 SE	UM ± 1 SE
Conductivity (μS/cm)	502.2 ± 140.2	168.7 ± 26.1	38.8 ± 4.4
pH	8.8 ± 0.6	7.5 ± 0.1	7.3 ± 0.1
Temperature (°C)	7.7 ± 0.2	8.5 ± 1.2	7.9 ± 0.3
DO (mg/l)	10.9 ± 0.3	10.9 ± 0.7	10.9 ± 0.2
**METALS (μg/l)**
[Cu]	0.5 ± 0.04	1.5 ± 0.1	0.5 ± 0.01
[Al]	92.3 ± 1.4	102.1 ± 1.8	95.7 ± 2.7
[Fe]	12.3 ± 1.1	42.3 ± 6.8	33.0 ± 10.4
[Si]	1703 ± 73	3176 ± 608	2278 ± 134
**CATIONS (mg/l)**
[Ca^2+^]	38.1 ± 9.0	14.0 ± 2.4	3.8 ± 0.8
[Mg^2+^]	27.4 ± 4.9	9.1 ± 1.8	1.9 ± 0.6
[K^+^]	1.1 ± 0.1	0.9 ± 0.02	0.9 ± 0.01
[Na^+^]	5.1 ± 1.5	1.6 ± 0.01	1.3 ± 0.01
**NUTRIENTS (μM)**
[TDN]	7.7 ± 0.7	17.9 ± 2.6	7.3 ± 1.2
[${\text{NO}}_{\text{3}}^{-}$]	2.8 ± 0.9	1.1 ± 0.1	4.3 ± 1.4
[TDN-${\text{NO}}_{\text{3}}^{-}$] = DON	5.1 ± 1.0	16.8 ± 2.6	3.0 ± 0.8
[${\text{NO}}_{\text{3}}^{-}$/TDN]	0.3 ± 0.1	0.1 ± 0.01	0.6 ± 0.1
[TDP]	0.2 ± 0.01	0.1 ± 0.01	0.2 ± 0.02
[TDN/TDP]	54.8 ± 5.9	128.6 ± 19.0	53.8 ± 11.4
**BASAL RESOURCES**
DOC (mg/l)	2.8 ± 0.2	8.3 ± 0.9	2.1 ± 0.2
Benthic Chl-*a* (μg/cm^2^)	0.3 ± 0.3	0.8 ± 1.1	0.2 ± 0.1

**Figure 2:**
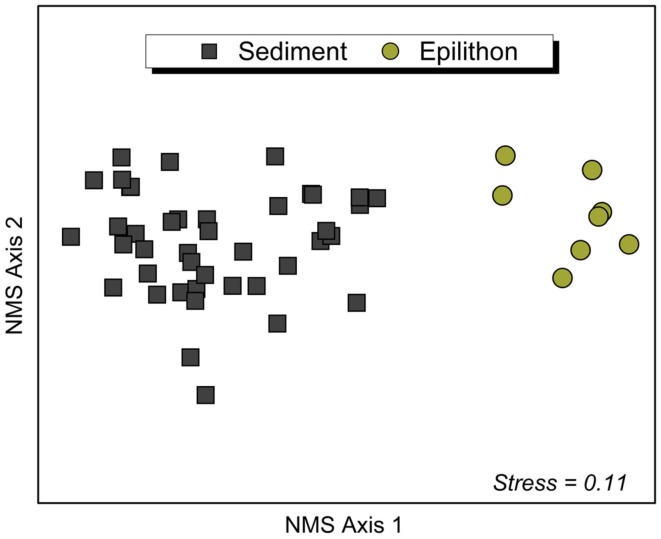
**Non-metric multidimensional scaling (NMS) ordination (first and second of the 3-dimensional solution) of stream sediment bacterial communities (left side, *n* = 42) and epilithon bacterial communities (right side, *n* = 8) based on pairwise similarity estimates (Bray–Curtis)**. Points that are close together represent communities with similar bacterial community composition based on the T-RFLP method. The associated normal stress value of the ordination is 0.11, indicating a good approximation of the overall structure of the data in multivariable space.

Non-metric multidimensional scaling and associated ANOSIM analysis of T-RFLP fragments confirmed differences in bacterial community composition between lithology and habitat. The composition of bacterial communities was significantly different between sediment and epilithon habitats (ANOSIM Global *R *= 0.98; *P *< 0.0001), with communities in each habitat clustering together regardless of the lithology of the catchment (Figure 2). The number of phylotypes (i.e., restriction fragments or T-RFs) in sediment samples ranged from 19 to 69 (mean: 51) with an average of 51 for CS, 54 for NC, and 50 for UM. The number of T-RFs in rock biofilm epilithon samples was higher and ranged from 66 to 99 (mean: 79) with an average of 72 for CS, 86 for NC, and 75 for UM.

**Figure 3:**
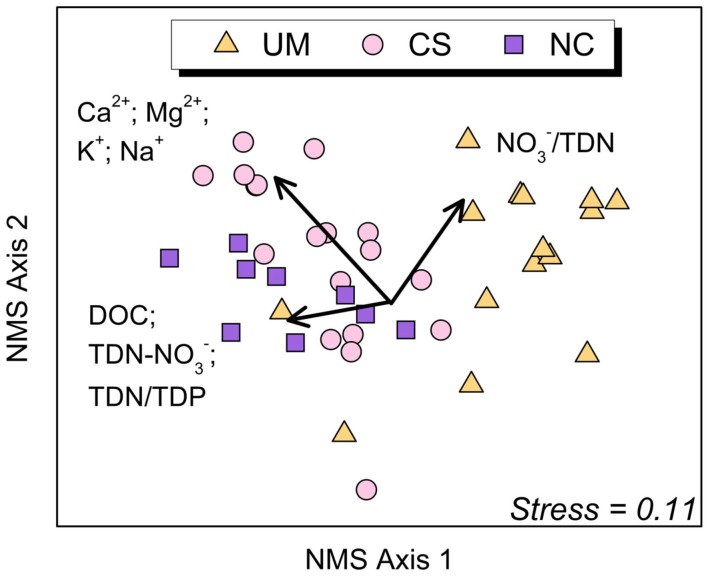
**Non-metric multidimensional scaling (NMS) ordination (first and second of the 3-dimensional solution) of stream sediment bacterial communities (*n* = 42) based on pairwise similarity estimates (Bray–Curtis)**. Points that are close together represent communities with similar bacterial community composition based on the T-RFLP method. Groupings show significant (ANOSIM Global *R *= 0.40; *P *< 0.001) separation by lithology (orange triangles = Ultramafic (UM); pink circles = Complex Sedimentary (CS); purple squares = Non-carbonate (NC). The associated normal stress value of the ordination is 0.11, indicating a good approximation of the overall structure of the data in multivariable space. Significant (*P* < 0.005), Bonferonni adjusted biogeochemical variables were overlaid (arrows) showing the degree of correlation with sediment data. Abbreviations: TDN-${\text{NO}}_{\text{3}}^{-}$ (total dissolved nitrogen minus nitrate, an indication of dissolved organic nitrogen); TDN/TDP (ratio of total dissolved nitrogen to total dissolved phosphorus); DOC (dissolved organic carbon); Base Cations = Ca^2+^, Mg^2+^, K^+^, and Na^+^; and ${\text{NO}}_{\text{3}}^{-}$/TDN (ratio of nitrate to total dissolved nitrogen).

Bacterial communities in the 42 sediment samples clustered by catchment lithology (ANOSIM Global *R *= 0.40; *P *< 0.001; Figure 3). Pairwise comparisons indicate that sediment communities in ultramafic UM streams were significantly different from those in NC and CS streams (UM vs. CS: *R* = 0.50, *P *< 0.0001; UM vs. NC: *R* = 0.55, *P *< 0.001; and NC vs. CS: *R* = 0.06, *P* = 0.2). Vector analysis indicated that DOC, base cations, TDN/TDP, TDN-${\text{NO}}_{\text{3}}^{-}$ (DON), and the proportion of ${\text{NO}}_{\text{3}}^{-}$/TDN were positively correlated with Axis 1 NMS sites scores (Bonferroni-corrected *P *> 0.05 in all cases; Figure 3). The UM and NC communities are separated along Axis 1 of the NMS, suggesting that the key biogeochemical differences (e.g., DOC, TDN/TDP, and TDN-${\text{NO}}_{\text{3}}^{-}$) may be driving these differences in community composition. Though not shown here, we also detected differences (ANOSIM Global *R* = 0.415, *P* = 0.048) by lithology with the limited epilithon samples (*n* = 2–4 per lithology).

Samples with the greatest difference in microbial communities based on T-RFLP patterns were selected for 16S rRNA gene cloning and sequencing from each of the three lithologies (two NC, two UM, and one CS; indicated on Figure 1). Epilithon samples were not analyzed from CS streams because after analyzing one CS sediment library we decided to compare the two most starkly contrasting lithologies (NC and UM). The number of clones for each of these nine libraries ranged from 33 to 87. Sequences were nearly full length extending from *Escherichia coli* positions 28–1491.

**Figure 4:**
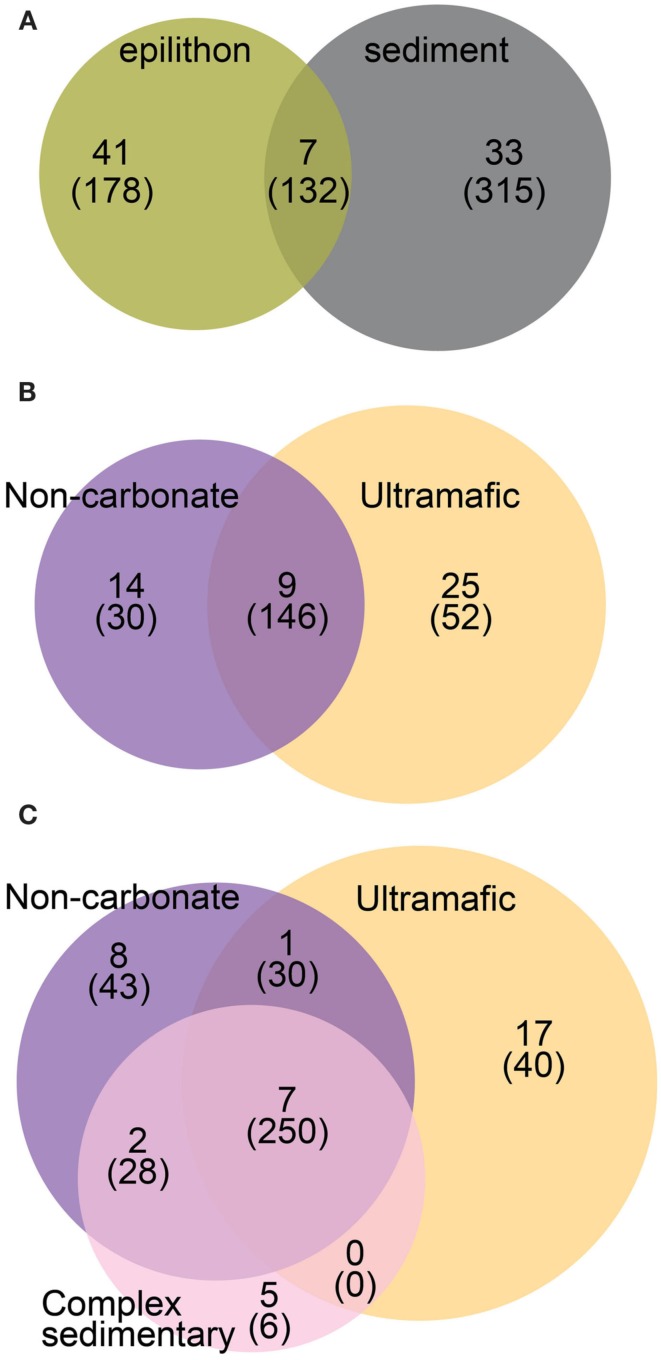
**Venn diagrams representing the overlap between samples at the following levels: (A) All OTUs grouped by habitat; (B) OTUs from epilithon samples grouped by lithology, and (C) OTUs from sediment samples grouped by lithology**. Numbers represent OTUs and parenthetical numbers represent sequences.

**Figure 5:**
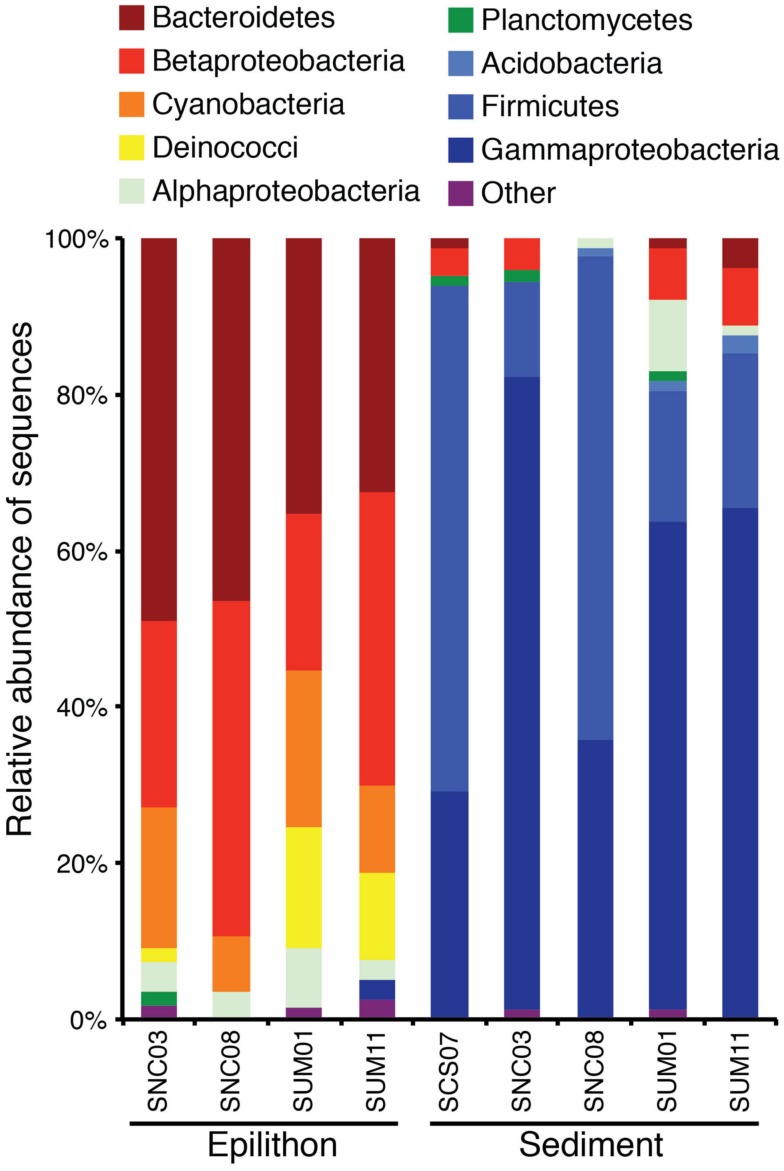
**Phylogenetic composition of clone sequences from stream epilithon and sediment samples in the non-carbonate (SNC), complex sedimentary (SCS), and ultramafic (SUM) lithologies expressed as relative abundance within each library**.

Of the 81 bacterial OTUs identified in all clone libraries, most were only found in one of the two habitats (Figure 4A). There were seven, nine, and seven cosmopolitan OTUs respectively, in sediment vs. epilithon samples (Figure 4A), epilithon samples by lithology (Figure 4B), and sediment samples by lithology (Figure 4C). There were fewer OTUs that were specific to a lithology while there were more that were specific to stream habitat type. The phylogeny of clone library sequences was different between sediment and epilithon samples (Figure 5). Sediment communities were dominated by Gammaproteobacteria (55%) belonging to several different orders, and Firmicutes in the order Bacillales (35%). In contrast, epilithon communities were dominated by Bacteroidetes (41%), Betaproteobacteria (31%), and Cyanobacteria (14%). These communities were consistent within habitat types, except that epilithon in UM streams supported a larger fraction of Deinococci, and sediments in ultramafic streams included Alphaproteobacteria in the order Rhizobiales. All of the taxa detected in the sediment and epilithon samples are widely distributed in the environment, including soil, sediments, seawater, and freshwater.

## Discussion

We hypothesized that inherent lithological characteristics of the Noatak National Preserve would impart a unique chemical composition to stream water, which in turn would introduce differences in bacterial community composition among NC, UM, and CS streams. Streams sampled were geomorphically similar in order, size, slope, and dominant benthic substrate type (cobble and gravel), yet the bacterial communities in these streams differed systematically according to biogeochemical characteristics of the stream water (e.g., DOC, nutrients, and base cations), which is likely a reflection of material inputs from the surrounding lithology and subsequent in-stream modification by biological activity.

Not surprisingly, bacterial communities in stream beds differed primarily by habitat type within streams (sediment vs. epilithon), and secondarily by the lithology of the upstream catchment. This latter observation suggests that the basic lithology of terrestrial environments influences not just the physical and aqueous biogeochemical structure of the environment, but the community composition of microorganisms that inhabit these environments. Sediment communities clustered by lithology with a high degree of separation between UM and NC communities and only partial separation between NC and CS. These trends mirror stream biogeochemistry across the three lithologies in that UM and NC has significantly different biogeochemical characteristics, while the NC and CS are similar. These results add stream sediment and epilithon to a small but growing list of habitats in which lithology of parent material correlates with microbial community composition. This list includes soils (Dunbar et al., 2000; Oline, 2006), glaciers (Skidmore et al., 2005), groundwater, and substratum (Takai et al., 2003).

Vector analysis indicates that certain biogeochemical variables explain NMS ordination of T-RFLP data (Figure 3), although causal relationships can only be inferred. Specifically, we observed a positive correlation between base cations and sediment communities from streams with CS lithology, suggesting bacterial community structure may be influenced by the high base cation concentration in CS streams, whereas the scarcity of cations in NC and UM streams may alter the composition of resident bacterial communities. Notably, calcium and magnesium are well known to enhance bacterial adhesion to substrates within the exopolysaccharide matrix of biofilms (Geesey et al., 2000), which may facilitate a niche for a more stable bacterial community in the CS lithology.

Other constituents such as DOC, TDN-${\text{NO}}_{\text{3}}^{-}$ (dissolved organic nitrogen), and TDN/TDP were found in highest concentrations in the NC streams, and correlated with NC community composition. Also, the percent proportion ${\text{NO}}_{\text{3}}^{-}$ of TDN was highest in UM streams, and was positively correlated with Axis 1 and 2 (UM community, Figure 3). DOC and TDN were lowest in streams of the CS and UM lithologies and highest in streams of the NC lithology. These trends further suggest that bacterial community composition may be influenced by the abundance or scarcity of resources, a likely consequence of surrounding lithology, soil type, and vegetation cover. Jorgenson et al. (2009) showed NC soils contain higher available soil phosphorus compared to UM soils, suggesting an interaction between vegetation and soils that may influence the nutrient content of soil water to streams in the Noatak region. The NC lithology supports relatively productive vegetation on land, which potentially correlates with higher available phosphorus in soil waters entering the NC streams; this would lead to increased uptake of ${\text{NO}}_{\text{3}}^{-}$, reduced stream water ${\text{NO}}_{\text{3}}^{-}$ content, and higher apparent productivity. Conversely, the lack of vegetation in the barren UM lithology may be an indication of the extreme phosphorus limitation of UM streams (indicated by the higher nitrogen concentrations) perhaps due to reduced phosphorus delivery to streams. These streams, in particular the UM streams, are phosphorus limited as are many streams on the North Slope of Alaska (Peterson et al., 1993; Bowden et al., 1994). This important landscape control appears to impart a biogeochemical fingerprint to waterways that shape the resident microbial communities within the streams bounded by unique lithologies.

Higher order controls on microbial community composition such as landscape and climate have been observed in studies that investigated the influence of microbial activity on redox chemistry and mineral processes in natural environments (Nealson and Stahl, 1997; Ehrlich, 1998). Furthermore, other studies have determined that microbial community composition can be correlated with observed aqueous geochemistry in subglacial chemical weathering (Skidmore et al., 2005), stream conductivity, and hydrology (Zeglin et al., 2011), stream water pH, quality of fine benthic organic matter, and quantity of DOC and nitrogen in stream water (Fierer et al., 2007), seasonal changes in temperature, nutrient availability, and light in estuarine biofilms (Moss et al., 2006), and flow heterogeneity (Singer et al., 2010). This study adds to the limited body of work demonstrating landscape influences on microbial community composition.

Clone libraries of 16S rRNA gene sequences confirmed the results of T-RFLP analysis concerning the discrimination of community composition by habitat and lithology and permitted the identification of bacterial taxonomic composition at the stream reach and landscape scale. Figure 5 shows that at the phylum and class levels, microbial communities are unique to each habitat type, but show substantial overlap across the two contrasting lithologies within habitat type. Thus, differences in community composition are more pronounced at the habitat scale than at the lithology scale. This difference was also reflected in the average number of phylotypes (T-RFs), which were greater in epilithon than sediment indicating a difference in taxonomic richness at the habitat scale. This result is likely due to the heterotrophic nature of the sediment environment and autotrophic nature of the epilithon environment. Differences between sediment and epilithon communities could also result from different hydrologic stressors. For example, varying flow regimes alter sediment structure via erosion and the redistribution of bacteria, exposing them to different environmental conditions (Hullar et al., 2006). While the epilithic community is not as likely to experience the same degree of disturbance as that found in the sediment, differences in hydrodynamic conditions are known to influence the structure and activity of epilithic biofilms (Battin, 2000; Battin et al., 2003).

Our results are similar to those reported by Hullar et al. (2006), who sampled headwater streams in southeast Pennsylvania and found differences between sediment and rock biofilm communities at the class-level. However, they differ from Hullar et al. (2006) in that we detected a high abundance of Cyanobacteria (14%) exclusively in rock biofilm samples whereas they found that Cyanobacteria comprised the majority (40%) of their sediment-derived sequences and a smaller proportion (25%) of the epilithic-derived sequences. The dominance of Cyanobacteria in the Hullar et al. (2006) sediment samples may be due to the more eutrophic nature of temperate streams they studied compared to the pristine arctic systems we sampled for this study. Moreover, we may have sampled at a greater depth into the sediment layer where light does not penetrate, thus explaining why photosynthetic Cyanobacteria in our sediment samples were not detected.

Ribosomal database project II Classifier was used to identify matches to clones at the phylum and order and class levels when possible. Many members of these phyla from the sediment samples: Acidobacteria; Firmicutes; and Proteobacteria (class: Gammaproteobacteria), are known heterotrophs and have been previously isolated from similarly classified pristine stream bed sediments in forested watersheds (Halda-Alija and Johnston, 1999). Orders within these phyla include: Aeromonadales, Pseudomonadales, and Xanthomonadales, all containing members that are obligately aerobic while Enterobacteriales and Bacillales members are facultatively anaerobic. Specializations of these groups include Enterobacteriaceae species having the ability to reduce nitrate to nitrite and Paenibacillaceae, a nitrogen-fixing group. Interestingly, Enterobacteriaceae were dominant in the relatively high ${\text{NO}}_{\text{3}}^{-}$ waters of the UM lithology and Paenibacillaceae were found to be dominant in the CS clone library where nitrate and TDN values were exceptionally low in CS streams, potentially explaining the persistence of a nitrogen-fixing bacterium.

Clones from the epilithon samples include the following phyla and classes: Bacteriodetes; Betaproteobacteria; Cyanobacteria; Deinicocci; Alphaproteobacteria; and Planctomycetes. Order members within these phyla (Sphingobacteriales, Burkholderiales, Deinicoccales, and Sphingomonadales) are varied in their function (chemoorganotrophic as well as obligately aerobic). Clones belonging to Sphingomonadales were found exclusively associated with the epilithic community and members of this Order have been isolated from a range of environments, including ultraoligotrophic waters, in which certain species (e.g., *S. alaskensis*) have been shown to possess physiological characteristics adapted to very low carbon substrate concentrations (Eiler et al., 2003). The presence of Sphingomonadales in epilithon samples may indicate lower availability of carbon sources for bacterial metabolism in this habitat; in contrast to the high loads of particulate and dissolved organic matter associated with stream sediment habitats that are influenced by upwelling areas from the hyporheic zone (Sobczak and Findlay, 2002).

In the past decade, studies on the taxonomic, phylogenetic, and physiological diversity of prokaryotes have begun to provide more comprehensive information about microbial communities and their natural environments, and in particular, whether microbes exhibit biogeographical patterns. Structural geographic patterns detected in microbial communities within stream ecosystems have been attributed to the following factors: geographic distance (<10 km) and connectivity between lakes and streams (Crump et al., 2007); biome-level control in low-order streams (Findlay et al., 2008); variation of chemical characteristics in streams across the southeastern and Midwestern USA (Gao et al., 2005); and landscape-level controls on streams due to biogeochemical factors (Fierer et al., 2007). In general, very few studies have focused on low-order streams (Hullar et al., 2006; Findlay et al., 2008) and none have included streams arising from catchments of single, uniform lithologies, as we have done in this study.

Our results suggest that there are differences in bacterial community composition across differing lithologies that can be related to large-scale linkages between streams and the terrestrial environment and parent material in which they are embedded. In turn, this relationship is reflected in differences in resource availability. Furthermore, the resident microorganisms of sediment and epilithon habitats are composed of significantly different bacterial taxa, indicating the presence of specialized ecological niches at the small-scale within stream ecosystems. Our study of arctic streams using T-RFLP and 16S rRNA gene sequencing indicates that bacterial community composition is influenced by lithological characteristics across the landscape as well as physical characteristics of habitat within an individual stream ecosystem.

## Conflict of Interest Statement

The authors declare that the research was conducted in the absence of any commercial or financial relationships that could be construed as a potential conflict of interest.

## Footnotes

^1^http://rdp.cme.msu.edu/

## References

[B1] Ashelford K. E. Chuzhanova N. A. Fry J. C. Jones A. J. Weightman A. J. (2005). At least 1 in 20 16S rRNA sequence records currently held in public repositories is estimated to contain substantial anomalies. Appl. Environ. Microbiol. 71, 7724–7736 10.1128/AEM.71.12.7724-7736.2005 16332745 PMC1317345

[B2] Autio R. (1998). Response of seasonally cold-water bacterioplankton to temperature and substrate treatments. Estuar. Coast. Shelf Sci. 46, 465–474 10.1006/ecss.1997.0282

[B3] Battin T. J. (2000). Hydrodynamics is a major determinant of streambed biofilm activity: from the sediment to the reach scale. Limnol. Oceanogr. 45, 1308–1319 10.4319/lo.2000.45.6.1308

[B4] Battin T. J. Kaplan A. Newbold D. Cheng X. Hansen C. (2003). Effects of current velocity on the nascent architecture of stream microbial biofilms. Appl. Environ. Microbiol. 69, 5443–5452 10.1128/AEM.69.9.5443-5452.2003 12957933 PMC194943

[B5] Bowden W. B. Finlay J. C. Maloney P. E. (1994). Long-term effects of PO4 fertilization on the distribution of bryophytes in an arctic river. Freshw. Biol. 32, 445–454 10.1111/j.1365-2427.1994.tb01138.x

[B6] Bray J. R. Curtis J. T. (1957). An ordination of the upland forest communities of Southern Wisconsin. Ecol. Monogr. 27, 325–349 10.2307/1942268

[B7] Brown M. P. S. (2000). “Small subunit ribosomal RNA modeling using stochastic context-free grammars,” in Proceedings of the Eighth International Conference on Intelligent Systems for Molecular Biology, La Jolla, 57–66 10977066

[B8] Caporaso G. Kuczynski J. Stombaugh J. Bittinger K. Bushman F. Costello E. Fierer N. Pena A. Goodrich J. Gordon J. Huttley G. Kelley S. Knights D. Koenig J. Ley R. Lozupone C. Mcdonald D. Muegge B. Pirrung M. Reeder J. Sevinsky J. Turnbaugh P. Walters W. Widmann J. Yatsunenko T. Zaneveld J. Knight R. (2010). QIIME allows analysis of high-throughput community sequencing data. Nat. Methods 7, 335–336 10.1038/nmeth.f.303 20383131 PMC3156573

[B9] Clarke K. R. (1993). Non-parametric multivariate analyses of changes in community structure. Aust. J. Ecol. 18, 117–143 10.1111/j.1442-9993.1993.tb00438.x

[B10] Clarke K. R. Green R. H. (1988). Statistical design and analysis for a “biological effects” study. Mar. Ecol. Prog. Ser. 46, 213–226 10.3354/meps046213

[B11] Cole J. R. Chai B. Farris J. Wang Q. Kulam-Syed-Mohideen A. S. Bandela A. M. Cardenas E. Garrity G. M. Tiedje J. M. (2007). The ribosomal database project (RDP-II): introducing myRDP space and quality controlled public data. Nucleic Acids Res. 35, D169–D172 10.1093/nar/gkm415 17090583 PMC1669760

[B12] Crump B. C. Adams H. E. Hobbie J. E. Kling G. W. (2007). Biogeography of bacterioplankton in lakes and streams of an Arctic tundra catchment. Ecology 88, 1365–1378 10.1890/06-0387 17601129

[B13] Crump B. C. Kling G. W. Bahr M. Hobbie J. E. (2003). Bacterioplankton community shifts in an Arctic lake correlate with seasonal changes in organic matter source. Appl. Environ. Microbiol. 69, 2253–2268 10.1128/AEM.69.4.2253-2268.2003 12676708 PMC154827

[B14] Denaro R. D’auria G. Di Marco G. Genovese M. Troussellier M. Yakimov M. M. Giuliano L. (2005). Assessing terminal restriction fragment length polymorphism suitability for the description of bacterial community structure and dynamics in hydrocarbon-polluted marine environments. Environ. Microbiol. 7, 78–87 10.1111/j.1462-2920.2004.00685.x 15643938

[B15] Deslippe J. R. Egger K. N. Henry G. H. R. (2005). Impacts of warming and fertilization on nitrogen-fixing microbial communities in the Canadian High Arctic. FEMS Microbiol. Ecol. 53, 41–50 10.1016/j.femsec.2004.12.002 16329928

[B16] Drever J. I. (2002). The Geochemistry of Natural Waters: Surface and Groundwater Environments. Englewood Cliffs, NJ: Prentice-Hall, Inc.

[B17] Dunbar J. Lawrence T. O. Kuske C. R. (2000). Assessment of microbial diversity in four Southwestern United States soils by 16S rRNA gene terminal restriction fragment analysis. Appl. Environ. Microbiol. 66, 2943–2950 10.1128/AEM.66.7.2943-2950.2000 10877790 PMC92095

[B18] Dunbar J. Ticknor L. O. Kuske C. R. (2001). Phylogenetic specificity and reproducibility and new method for analysis of terminal restriction fragment profiles of 16S rRNA genes from bacterial communities. Appl. Environ. Microbiol. 67, 190–197 10.1128/AEM.67.1.190-197.2001 11133445 PMC92545

[B19] Ehrlich H. I. (1998). Geomicrobiology: its significance for geology. Earth Sci. Rev. 45, 45–60 10.1016/S0012-8252(98)00034-8

[B20] Eiler A. Laggenheder S. Bertilsson S. Tranvik L. J. (2003). Heterotrophic bacterial growth efficiency and community structure at different natural organic carbon concentrations. Appl. Environ. Microbiol. 69, 3701–3709 10.1128/AEM.69.7.3701-3709.2003 12839735 PMC165184

[B21] Fierer N. Morse J. L. Berthrong S. T. Bernhardt E. S. Jackson R. B. (2007). Environmental controls on the lanscape-scale biogeography of stream bacterial communites. Ecology 88, 2162–2173 10.1890/0012-9658(2007)88[1336:FAPFMI]2.0.CO;2 17918395

[B22] Findlay R. H. Yeates C. Hullar M. A. J. Stahl D. S. Kaplan L. A. (2008). Biome-level biogeography of streambed microbiota. Appl. Environ. Microbiol. 74, 3014–3021 10.1128/AEM.01809-07 18378660 PMC2394931

[B23] Findlay S. Sinsabaugh R. (2006). Large-scale variation in subsurface stream biofilms: a cross-regional comparison of metabolic function and community similarity. Microb. Ecol. 52, 491–500 10.1007/s00248-006-9095-z 16909347

[B24] Flinn M. Bowden W. Peterson B. Luecke C. Balser A. Allen A. Larouche J. (2009). “The Influence of Lithology on Physical, Chemical, and Biological Characteristics of Headwater Streams in the Feniak Lake Region, Noatak National Preserve, Alaska.” in Final Report to the National Park Service. Fairbanks: Arctic Network

[B25] Galand P. E. Lovejoy C. Vincent W. F. (2006). Remarkably diverse and contrasting archaeal communities in a large arctic river and the coastal Arctic Ocean. Aquat. Microb. Ecol. 44, 115–126 10.3354/ame044115

[B26] Gao X. Olapade O. A. Leff L. G. (2005). Comparison of benthic bacterial community composition in nine streams. Aquat. Microb. Ecol. 40, 51–60 10.3354/ame040051

[B27] Garneau M. E. Vincent W. F. Alonso-Saez L. Gratton Y. Lovejoy C. (2006). Prokaryotic community structure and heterotrophic production in a river-influenced coastal arctic ecosystem. Aquat. Microb. Ecol. 42, 27–40 10.3354/ame042027

[B28] Geesey G. G. Wigglesworth-Cooksey B. Cooksey K. E. (2000). Influence of calcium and other cations on surface adhesion of bacteria and diatoms: a review. Biofouling 15, 195–205 10.1080/08927010009386310 22115304

[B29] Gower J. C. (1971). A general coefficient of similarity and some of its properties. Biometrics 27, 857–871 10.2307/2528823

[B30] Gutell R. R. Lee J. C. Cannone J. J. (2002). The accuracy of ribosomal RNA comparative structure models. Curr. Opin. Struct. Biol. 12, 301–310 10.1016/S0959-440X(02)00339-1 12127448

[B31] Halda-Alija L. Johnston T. C. (1999). Diversity of culturable heterotrophic aerobic bacteria in pristine stream bed sediments. Can. J. Microbiol. 45, 879–884 10.1139/w99-081 10907425

[B32] Hall R. O. Baker M. A. Arp C. D. Koch B. J. (2009). Hydrologic control of nitrogen uptake, storage, and export in a mountain stream. Limnol. Oceanogr. 54, 2128–2142 10.4319/lo.2009.54.3.0880

[B33] Hullar M. Kaplan L. A. Stahl D. A. (2006). Recurring seasonal dynamics of microbial communities in stream habitats. Appl. Environ. Microbiol. 72, 713–722 10.1128/AEM.72.1.713-722.2006 16391111 PMC1352240

[B34] Hynes H. B. N. (1975). The Stream and Its Valley. Verhandlungen der Internationalen Vereinigun fur Theoretische and Angewandte Limnologie 19, 1–15

[B35] Jenny H. (1980). The Soil Resource: Origin and Behavior. Berlin: Springer-Verlag, 37

[B36] Jorgenson M. T. Swanson D. K. Macander M. (2002). “Landscape-level mapping of ecological units for the Noatak National Preserve, Alaska,” in Final Report to the National Park Service. Fairbanks: Arctic Network

[B37] Jorgenson T. Center N. R. P. Abr I. (2009). An Ecological Land Survey and Landcover Map of the Arctic Network. U. S. Department of the Interior, National Park Service, Natural Resource Program Center

[B38] Kaplan L. Bott T. (1989). Diel fluctuations in bacterial activity on streambed substrata during vernal algal blooms: effects of temperature, water chemistry, and habitat. Limnol. Oceanogr. 34, 718–733 10.4319/lo.1989.34.4.0718

[B39] Liu W. Marsh T. L. Cheng H. Forney L. J. (1997). Characterization of microbial diversity by determining terminal restriction fragment length polymorphisms of gene encoding 16S rRNA. Appl. Environ. Microbiol. 63, 4516–4522 9361437 10.1128/aem.63.11.4516-4522.1997 PMC168770

[B40] Ludwig W. Strunk O. Westram R. Richter L. Meier H. Yadhukumar Buchner A. Lai T. Steppi S. Jobb G. Förster W. Brettske I. Gerber S. Ginhart A. W. Gross O. Grumann S. Hermann S. Jost R. König A. Liss T. Lüßmann R. May M. Nonhoff B. Reichel B. Strehlow R. Stamatakis A. Stuckmann N. Vilbig A. Lenke M. Ludwig T. Bode A. Schleifer K. H. (2004). ARB: a software environment for sequence data. Nucleic Acids Res. 32, 1363–1371 10.1093/nar/gkh293 14985472 PMC390282

[B41] Milner A. M. Oswood M. W. Munkittrick K. R. (2005). “Rivers of Arctic North America,” in Rivers of North America, eds Benke A. Cushing C. (Burlington: Elsevier Academic Press).

[B42] Minchin P. (1990). DECODA: Database for Ecological Community Data. Canberra: Australian National University

[B43] Moss J. A. Nocker A. Lepo J. E. Snyder R. A. (2006). Stability and change in estuarine biofilm bacterial community diversity. Appl. Environ. Microbiol. 72, 5679–5688 10.1128/AEM.02773-05 16957182 PMC1563641

[B44] Nealson K. H. Stahl D. A. (1997). “Microorganisms and biogeochemical cycles: what can we learn from layered microbial communities?” in Geomicrobiology: Interactions Between Microbes and Minerals, eds Nealson K. H. Banfield J. F. (Washington, DC: Mineralogical Society of America), 5–34

[B45] Oline D. K. (2006). Phylogenetic comparisons of bacterial communities from serpentine and nonserpentine soils. Appl. Environ. Microbiol. 72, 6965–6971 10.1128/AEM.00690-06 16950906 PMC1636195

[B46] Peterson B. J. Deegan L. Helfrich J. Hobbie J. E. Hullar M. Moller B. Ford T. E. Hershey A. Hiltner A. Kipphut G. Lock M. A. Fiebig D. M. Mckinley V. Miller M. C. Vestal J. R. Ventullo R. Volk G. (1993). Biological responses of a tundra river to fertilization. Ecology 74, 653–672 10.2307/1940794

[B47] Rees G. N. Baldwin D. S. Watson G. O. Perryman S. Nielsen D. L. (2004). Ordination and significance testing of microbial community composition derived from terminal restriction fragment length polymorphisms: application of multivariate statistics. Antonie Van Leeuwenhoek 86, 339–347 10.1007/s10482-004-0498-x 15702386

[B48] Reysenbach A. L. Pace N. R. (1995) In Archaea: A Laboratory Manual, ed. Robb F. (Cold Spring Harbor, NY: Cold Spring Harbor Lab. Press), 101–107

[B49] Schloss P. D. Westcott S. L. Ryabin T. Hall J. R. Hartmann M. Hollister E. B. Lesniewski R. A. Oakley B. B. Parks D. H. Robinson C. J. Sahl J. W. Stres B. Thallinger G. G. Van Horn D. J. Weber C. F. (2009). Introducing mothur: open-source, platform-independent, community-supported software for describing and comparing microbial communities. Appl. Environ. Microbiol. 75, 7537–7541 10.1128/AEM.01541-09 19801464 PMC2786419

[B50] Singer G. A. Besemer K. Schmitt-Kopplin P. HÖdl I. Battin T. J. (2010). Physical heterogeneity increases biofilm resource use and its molecular diversity in stream mesocosms. PLoS ONE 5, e9988 10.1371/journal.pone.0009988 20376323 PMC2848676

[B51] Skidmore M. Anderson S. P. Sharp M. Foght J. Lanoil B. D. (2005). Comparison of microbial community compositions of two subglacial environments reveals a possible role for microbes in chemical weathering processes. Appl. Environ. Microbiol. 71, 6986–6997 10.1128/AEM.71.11.6986-6997.2005 16269734 PMC1287656

[B52] Slavik K. Peterson B. J. Deegan L. A. Bowden W. B. Hershey A. E. Hobbie J. E. (2004). Long-term responses of the Kuparuk river ecosystem to phosphorus fertilization. Ecology 85, 939–954 10.1890/02-4039

[B53] Sobczak W. V. Findlay S. (2002). Variation in bioavailability of dissolved organic carbon among stream hyporheic flowpaths. Ecology 83, 3194–3209 10.1890/0012-9658(2002)083[3194:VIBODO]2.0.CO;2

[B54] Takai K. Mormile M. R. Mckinley J. P. Brockman F. J. Holben W. E. Kovacik W. P. Fredrickson J. K. (2003). Shifts in archaeal communities associated with lithological and geochemical variations in subsurface Cretaceous rock. Environ. Microbiol. 5, 309–320 10.1046/j.1462-2920.2003.00421.x 12662178

[B55] Van Hannen E. J. Mooij W. Van Agterveld M. P. Gons H. J. Laanbroek H. J. (1999). Detritus-dependent development of the microbial community in an experimental system: qualitative analysis by denaturing gradient gel electrophoresis. Appl. Environ. Microbiol. 65, 2478–2484 10347030 10.1128/aem.65.6.2478-2484.1999 PMC91365

[B56] Wang Q. Garrity G. M. Tiedje J. M. Cole J. R. (2007). Naïve Bayesian classifier for rapid assignment of rRNA sequences into the new bacterial taxonomy. Appl. Environ. Microbiol. 73, 5261–5267 10.1128/AEM.00062-07 17586664 PMC1950982

[B57] Whittaker R. H. (1960). Vegetation of the Siskiyou mountains, Oregon and California. Ecol. Monogr. 30, 279–338 10.2307/1948435

[B58] Zeglin L. H. Dahm C. N. Barrett J. E. Gooseff M. N. Fitpatrick S. K. Takacs-Vesbach C. D. (2011). Bacterial community structure along moisture gradients in the parafluvial sediments of two ephemeral desert streams. Microb. Ecol. 61, 543–556 10.1007/s00248-010-9782-7 21153024

